# Relationship between body composition and upper limb physical fitness among Chinese students: 4-Year longitudinal follow-up and experimental study

**DOI:** 10.3389/fphys.2023.1104018

**Published:** 2023-03-03

**Authors:** Qingmei Wang, Junwei Qian, Haoran Pan, Qianqian Ju

**Affiliations:** ^1^ Tianjin College, Beijing University of Science and Technology, Tianjin, China; ^2^ Department of Physical Education, Peking University, Beijing, China; ^3^ The School of Health Humanities, Peking University, Beijing, China; ^4^ School of Psychological and Cognitive Sciences, Peking University, Beijing, China

**Keywords:** students, body composition, BMI-body mass index, physical fitness, pull-up performance

## Abstract

**Background:** Recently, students’ fitness has been declining, and high physical fitness level is crucial in establishing optimal physical/mental health and academic performance. The purpose of this study was to explore the relationship between body composition and upper limb physical fitness and the specific aspects of low physical fitness level in Chinese students. Exploring the development and impact factors for upper limb physical fitness can provide a theoretical basis for the health management strategy of students.

**Methods:** Study 1 collected data from 183 male students over 4 years and used Hierarchical Linear Model (HLM) to explore the quadratic predictive role of body composition on upper limb physical fitness. To further explored which aspects of upper limb physical fitness were affected by body composition, study 2 conducted an experimental investigation among 42 male students, comparing different kinds of upper limb physical fitness within two different body composition groups.

**Results:** Studies found (1) from 2015 to 2018, students’ Body-Mass-Index (BMI) showed an upward trend, and BMI differences were significant from year to year. While the upper limb physical fitness showed a downward trend. There were significant differences in the number of pull-outs between 2015 and 2016, 2015 and 2017, and 2015 and 2018. (2) The quadratic term of BMI could predict the upper body physical fitness in the same year and the following year. That is, when BMI was medium, the upper body fitness of the same year and the following year was the best. (3) Chinese students with excellent body composition had greater grip strength, drape height and anaerobic power than those with average body composition.

**Conclusion:** In recent years, male students’ BMI has been increasing, and the upper body physical fitness has been decreasing. Furthermore, body composition can predict the upper body mass in the same year and the second year, and male students with better body composition also had greater grip strength, drape height and anaerobic power in their upper limbs.

## Background

Physical fitness is one of the important signs of health (Granger et al., 2017). Relative to students with lower physical fitness, students with higher physical fitness have more opportunity to participate in sport activities (Marttinen et al., 2018), lower risk of health problems (Kao et al., 2020), better cognitive function (Reigal et al., 2020) and higher quality of life (Marker et al., 2018; Bermejo-Cantarero et al., 2021).

However, the situation of students’ physical fitness is not optimistic. The trend of physical fitness among Chinese students was declining over the past years (Tian et al., 2016). Similar results have been found in other countries (Marttinen et al., 2018; Masanovic, et al., 2020a; Farooq et al., 2020). For example, Masanovic, et al., 2020a found a constant decline in strength and endurance through a systematic review among students from 14 countries. Furthermore, the reasons for the decline included not only weight increase (Zhang et al., 2018) and lifestyle changes (like increase in physical inactivity and screen time, Masanovic, et al., 2020a), but also governments’ policies regarding physical activity (Costa et al., 2017). In China, the government issued relevant policies to improve the physical fitness for young adults. For example, the government revised the “*National Physical Health Standards for Students (Revised in 2014)*” (http://sports.upc.edu.cn/2020/0629/c7653a307672/page.htm) to increase the proportion of physical fitness tests in the total score. Also, they issued “Healthy China 2030” and “China’s Education Modernization 2035” strategy to emphasize the importance of promoting the physical development for Chinese children and young adults.

For Chinese college students, after entering college, they invest less in physical education courses compared with other courses, so their participation in sports activities decreases with the increase of college grades (Ting et al., 2021). Here, we aim to investigate the development tendency of college students’ physical fitness and its influencing factor, improving our understanding of the situation of college students’ physical fitness. Considering previous studies have found a decline in physical fitness among students with their ages (Masanovic, et al., 2020b), we expected physical fitness to decline as college grades increase, and hypothesis 1a was proposed.

H1a: Students’ physical fitness gradually declined during the first and fourth years of college.

Recently, with the worldwide prevalence of obesity, body composition (such as Body-Mass-Index, BMI, calculated with individuals’ weight and height) has attracted more and more attention (Tomiyama, 2019). For example, a study published in Lancet (Wang et al., 2021) found the average BMI and obesity of Chinese adults have been steadily increasing from 2004 to 2018. Studies also found BMI was negatively correlated with muscle function (Bollinger, 2017) and physical fitness (Bulbrook et al., 2021). The development of upper limb strength may be limited for both the overweight people with large BMI, and the underweight people with low BMI because of lower muscle mass. Lopes et al. (2019) also found a quadratic relationship between BMI and physical fitness in Brazilian youth. However, in China, the non-linear relationship between body composition and physical fitness was not verified, and the casual relationship was also unclear. To explore the casual effect of body composition on upper limb physical fitness, we proposed hypothesis 1b.

H1b: The degree of healthier body composition would facilitate upper body physical fitness.

To evaluate hypotheses 1a and hypotheses 1b, through exploring the developmental trajectory of students’ physical fitness and the specific impact of body composition on physical fitness, study 1 conducted a longitudinal follow-up survey for 4 years (2015–2018) among male students from several universities in Beijing. The performance of pull-up was used as objective indicators of physical fitness, and BMI was measured as a body composition index. Analysis of variance (ANOVA) and Chi-square test were used to investigate the developmental tendency of upper limb physical fitness, and regression analysis and Hierarchical Linear Model (HLM) were further used to explore the predictive relationship between BMI and pull-up performances with 4-year follow-up data.

After exploring the predicted relationship between body composition and upper limb physical fitness, we examine whether the effect remained when body composition was measured more accurately, and what aspects of upper limb physical fitness were affected by body composition. For more accurate body composition measurement, Study 2 used GE dual-energy X-ray absorptiometry to measure body composition, and the measurements of body fat percentage and lean body mass percentage were better than BMI, which was often used in previous studies (Pan et al., 2021; Wang et al., 2021), because the relation between two measurements and the health degree was monotonic. In this way, we could easily interpret lower body fat percentage and higher lean body mass percentage as the reflections of more health.

To measure upper limb physical fitness more comprehensively, we selected 3 different indices, including 1) Handgrip strength, 2) upper body strength with Power Slap Test (Draper et al., 2011), and 3) anaerobic power with Wingate anaerobic test (Patton et al., 1985). Physical activity (e.g., pull-ups) is beneficial for improvement of upper body power performance (Sas-Nowosielski and Kandzia, 2020). With single-factor design, the independent variable of this study was body composition groups (athlete group vs. non-athlete group), and the dependent variables were 3 kinds of upper limb physical fitness. Hypothesis 2 was proposed.

H2: Body composition would be positively correlated with the upper limb physical fitness in grip strength, upper body strength and anaerobic power of upper limbs.

## Methods

### Study 1

#### Experimental procedure

In study 1, we accessed the data from Department of Physical Education in Peking University. Male freshmen enrolled in a university in Beijing in 2015 was recruited, and provided informed consent for all participants before participating. Pull-up and BMI data were recorded during mid-April or late October of each year during the study period from 2015 to 2018. We matched 332 student data according to their ID, excluded students who had not participated in the measurement for more than one year, and the data analysis included 183 of them. According to standard Chinese criteria (Liu et al., 2022), for 183 participants (16.31 < BMI< 42.61 kg/m2), 20 of them was thin (BMI <18.5), 122 was in normal weight (18.5 < BMI <23.9), 32 was overweight (24 < BMI <27.9), and 9 was obesity (BMI >28). We could not access the data for female because the universities provided data for our studies (Beijing, China) did not measure their pull-up performance.

#### Ethics approval and consent to participate

Study was conducted according to the guidelines of the declaration of Helsinki, and have been approved by the Committee for Protecting Human and Animal Subjects at School of Psychological and Cognitive Sciences in Peking University. Before participating in the study (2015), all participants were informed of the project contents, risks and benefits, and could withdraw at any time. The informed consents were obtained from all participants.

#### Measurements

***Numbers and grades of pull-ups*** The upper limb physical fitness was measured by numbers and grades of pull-ups. Number of pull-ups were measured in a Beijing university stadium using a pull-up machine from Siboyou Company. The tester jumps up and grabs the bar with both hands, arms hanging straight at shoulder width, and body still. Then, both arms pull up at the same time, pull up to the lower chin over the upper edge of the bar, the machine issued a drip slowly lowered the body to restore static, then one test completed (As shown in Supplementary Figure S1). The grades of pull-ups are divided according to Chinese *National Physical Health Standards for Students*: for freshmen and sophomores, 0–9 pull-ups are considered as fail, 10–14 as pass, 15–16 as good, and 17 or more as excellent. For juniors and seniors, 0–10 pull-ups are considered fail, 11–15 pass, 16–17 good, and 18 or more excellent.

***Body-Mass-Index (BMI)*** Body composition was measured by BMI. BMI value was calculated based on the “weight (kg)/height (m)^2”. Height and weight were tested in a university stadium in Beijing using a machine from Siboyou Company. The participant stands barefoot on the instrument with the dipstick resting on their heels, sacrum, and shoulders, eyes straight ahead. After standing on the instrument and keeping still, the height and weight will be recorded and displayed automatically.

#### Data analyses

R 4.1.0 and Mplus 8.3 were used for data analysis. The R software is used for data preprocessing. ANOVA, Chi-square test, and regression analysis were used for the pull-up and BMI results, and Hierarchical Linear Models were conducted with Mplus to explore the quadratic relationship between BMI and the following year’s pull-up performance.

### Study 2

#### Experimental procedure

Study 2 was conducted from September to October 2018, and 42 young male students from universities in Beijing were included and analyzed. The participants in study 2 were independent from those in Study 1. Among them, 21 students whose sports level reached grade two or above were randomly selected as the excellent physical composition students (Athlete Group), and their sports training years were 2.42 (±0.95) years on average. Meanwhile, 21 students whose sports level did not reach grade were randomly selected as the common body composition students (Non-athlete Group). The grade about sports level would be evaluated only after students participate in municipal and above level sport competitions and reaches the standard of General Administration of Sports in China (https://www.sport.gov.cn/n20001280/n20067662/n20067740/c23624534/content.html).

The results of the manipulation check showed that there were significant differences in body composition (body fat percentage and lean body weight percentage) between two groups, and the body fat percentage to lean body weight percentage of the athlete group were significantly lower than those of the non-athlete group (*ps*≤0.001), and the selection of the two groups of participants is effective (as shown in Table 1). All students had adequate sleep, no strenuous exercise, no metal graft in the body in 24 h before the test, and no upper limb injury in the last 6 months.

**TABLE 1:** Body composition measurement for the athlete group and the non-athlete group.

Body composition measurement	Athlete group (95% CI)	Non-athlete group (95% CI)	*F*	*p*	Cohen’s *d*
Body fat percentage (%)	8.49 ± 2.61 (7.30, 9.68)	12.94 ± 4.62 (10.60, 14.79)	14.892	<0.001	−1.186
Lean body mass percentage (%)	0.925 ± 0.026 (0.903, 0.927)	0.873 ± 0.046 (0.852, 0.894)	13.317	<0.001	1.392

#### Ethics approval and consent to participate

Study 2 was conducted according to the guidelines of the declaration of Helsinki, and have been approved by the Committee for Protecting Human and Animal Subjects at School of Psychological and Cognitive Sciences in Peking University (the approval number is #2018-10-01). Before participating in this study, all participants in each study were informed of the project contents, risks and benefits, and could withdraw at any time. The informed consents were obtained from all participants.

#### Measurements

***Body composition*** Body composition was measured by total body fat percentage and total lean body mass percentage. We scanned participants’ bodies using GE dual-energy X-ray absorptiometry. The body scan was carried out with medium speed and medium aim, and the combination of sector scan and dot beam scan. The DXA model was GE Lunar Prodigy, and the analysis software version was enCORE10.50.086. After scanning and analysis, the total body fat percentage and total lean body mass percentage would be calculated.

***Upper Limb Physical fitness*** We included grip strength test, upper power and upper limb anaerobic power measurement for upper limb physical fitness.

(1) ***Grip strength*** Participants were tested on their hand grip strength. Participants stood upright with their arms hanging down naturally. They held a grip meter with the palm inward and the dial outward tilted at 45°. Verbal encouragement was given to participants during the test. We measured grip strength on each side 3 times and recorded the best result.
(2) ***Upper body strength*** Power Slap Test was used to test upper body power performance (Draper et al., 2011; Sas-Nowosielski and Kandzia, 2020). We selected a vertical rock wall that was perpendicular to the ground and protruding, and installed two holding points of the same specifications on the rock wall. The holding points were 2.4 m above the ground and the distance between the two holding points was 45 cm (as shown in Supplementary Figure S2). The scale ruler was stuck in the middle of the holding points. During the test, participants grasped the fulcrum with both hands to make their bodies hang naturally. After stabilizing, they attempted their best to pull up and touch the scale with their hands and recorded the height distance. They repeated the test 3 times with 2-min intervals for rest. The best result would be counted into the statistics. We ruled out the effect of wingspan on test scores by defining the *Power Slap Index* as the high dangling touch score divided by wingspan.
(3) ***Anaerobic power of upper limbs*** Wingate anaerobic test was used (Patton et al., 1985). The test instrument was Monark 891E upper limb anaerobic power meter, and the resistance coefficient was set to 0.05 kP/kg (Kilopond ·kg-1BW). The test was conducted in a sitting position, with the seat adjusted so that the participant’s shoulders were parallel to the power generator axle. During the measurement, participants accelerated for 4 s under non-resistance load and then loaded resistance. After that, participants shook the crank at the fastest speed for 30 s. During this period, bicycle speed and number of turns were recorded every 5 s to calculate the output power.

#### Data analyses

IBM SPSS 24.0 were used for data analysis. ANOVA was conducted, and the effect size of Cohen’s *d* representative data was calculated, where 0.5 or above was considered as medium effect and 0.8 or above was considered as high efficiency (Gignac & Szodorai, 2016).

## Results

### Study 1

#### Pull-up performance declined and BMI increased year by year

**FIGURE 1:**
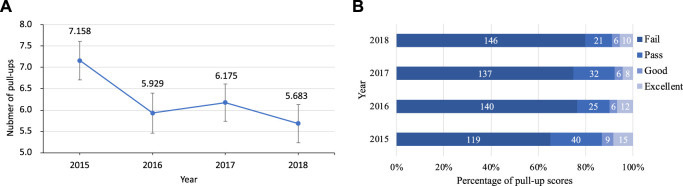
Change tendency of pull-ups from 2015 to 2018 **(A)**. Line chart for number of pull-ups, **(B)**. Bar chart for grades of pull-ups and numbers of students.

This study analyzed the number of students’ pull-ups and found that since 2015, the number of students’ pull-ups has shown a downward tendency. ANOVA was used to examine the number of pull-ups and found the main effect of years was significant (*F*_(180, 3)_ = 7.057, *p* < .001, *η* ^*2*^ = 0.037). Among them, there were significant differences between the numbers in 2015 and 2016 (*p* = 0.005), 2015 and 2017 (*p* = 0.031), 2015 and 2018 (*p* < 0.001). More details shown in Figure 1.

Besides, in the analysis of students’ pull-up grades, more than 60% of the students failed during the 4-year period, and the number of failed students increased with the increase of years. With Chi-square test, we found no significant difference in 4-year’ pull-up performance (*χ* ^^2^_*(9)*_ = 13.451, *p* = 0.143).

**FIGURE 2:**
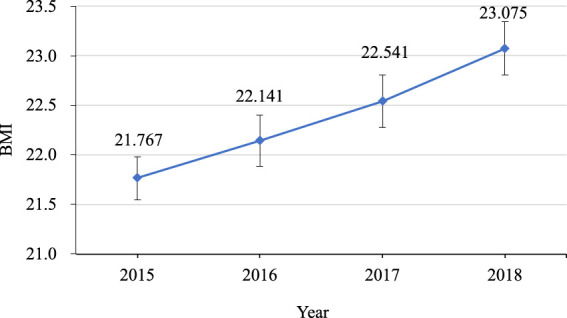
Line chart of BMI change tendency from 2015 to 2018.

BMI of students was also analyzed, and it was found that BMI was increasing year by year. ANOVA for BMI showed that the main effect of years was significant (*F*_(180, 3)_ = 45.418, *p* < .001, *η* ^*2*^ = 0.200), and the difference between each years was significant (*ps*≤0.027). More details shown in Figure 2.

#### The quadratic BMI predicted the number of pull-ups

In order to further explore the factors influencing the number of pull-ups, this study used regression analysis to explore the relationship between BMI and pull-ups. The results showed that the quadratic BMI significantly predicted the number of pull-ups in the same year (*β* = −0.337, R^^2^ = 0.118, *p* < .001).

**FIGURE 3:**
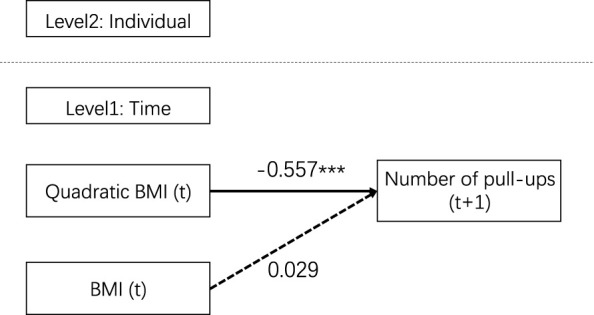
Hierarchical Linear Model for quadratic BMI predicting the number of pull-ups in the following year.

Further, HLM was used to explore the prediction effect of quadratic BMI on the number of pull-ups at t +1 from the individual level from 2015 to 2018 (see Figure 3 for details). The study established a two-level linear model (level 2: individual, level 1: time point of measurement), and all variables were averaged by groups. The results showed that the model fit well (AIC = 3169.933, BIC = 3191.418, *χ* ^^2^(*df*) = 38.616(2), RMSEA = 0.000, SRMR = 0.001, CFI = 1.000, TLI = 1.000). Quadratic BMI significantly predicted the number of pull-ups in the following year (*β* = −0.577, SE = 0.059, 95%CI = [-0.707, −0.406]), while BMI had no significant predictive effect (*β* = 0.029, SE = 0.046, 95%CI = [-0.088, 0.147]). That is, the number of pull-ups in the following year was greatest when BMI was in the middle range.

### Study 2

#### Manipulation check

ANOVA analysis was used for manipulation check. The analysis found that the body fat percentage of the **athlete group** was significantly lower than that of the non-athlete group (*p* < 0.001), and the lean body weight percentage was significantly higher than that of the non-athlete group (*p* < 0.001), that is, there were significant differences in the body composition of the two groups, as shown in Table 1.

#### Physical fitness between two groups with different body composition

The results showed that the handgrip strength score and index of the athlete group were significantly higher than that of the non-athlete group (*ps* < 0.019), and the score and index of Power Slap Test were significantly higher than that of the non-athlete group (*ps* < 0.001). The anaerobic performances of the upper limbs were also different for the two groups (*ps* < 0.018), as shown in Table 2. Among them, the most significant difference was in score and index for Power Slap Test (Cohen’s *d* = 3.318, 3.052), followed by average power/body weight in upper limb anaerobic performances (Cohen’s *d* = 1.718). The least difference was the power decay rate of the anaerobic power of the upper limbs (Cohen’s *d* = −0.662).

**TABLE 2:** Physical fitness results for athlete group and non-athlete group.

Indicators		Athlete group (95% CI)	Non-athlete group (95% CI)	*F*	*p*	Cohen’s *d*
Handgrip Strength	Left handgrip Strength	0.72 ± 0.09 (0.68, 0.77)	0.65 ± 0.08 (0.62, 0.69)	5.952	0.019	0.822
	Right handgrip Strength	0.78 ± 0.13 (0.72, 0.83)	0.68 ± 0.09 (0.65, 0.72)	8.062	0.007	0.894
	Index for Dominant hand	0.78 ± 0.12 (0.73, 0.84)	0.70 ± 0.08 (0.66, 0.73)	7.315	0.010	0.784
	Index for Non-dominant hand	0.72 ± 0.10 (0.67, 0.76)	0.65 ± 0.08 (0.61, 0.68)	6.419	0.015	0.773
Upper Body Strength	Score (cm)	100.76 ± 4.95 (98.5, 103.0)	68.59 ± 14.06 (62.2, 74.8)	98.23	<0.001	3.052
	Index (Score/Wingspan)	0.60 ± 0.03 (0.58, 0.61)	0.40 ± 0.08 (0.36, 0.44)	95.82	<0.001	3.31
Anaerobic Power of Upper Limbs	Peak Power (W)	466.58 ± 62.21 (438.3, 494.9)	417.45 ± 66.58 (387.2, 447.8)	6.105	0.018	0.763
	Average Power(W)	374.49 ± 43.06 (354.9, 394.1)	309.04 ± 58.66 (282.3, 335.7)	16.988	<0.001	1.272
	Minimum Power(W)	272.14 ± 34.32 (256.5, 287.8)	217.17 ± 52.08 (193.5, 240.9)	16.314	<0.001	1.246
	Peak Power/Weight (W·kg^−1^BW)	7.61 ± 1.00 (7.15, 8.06)	6.52 ± 0.89 (6.12, 6.93)	13.834	0.001	1.151
	Index (Average Power/Weight) (W·kg^−1^BW)	6.09 ± 0.58 (5.83, 6.36)	4.83 ± 0.86 (4.44, 5.22)	31.345	<0.001	1.718
	Power decay rate (%)	41.24 ± 6.59 (38.2, 44.2)	47.59 ± 11.85 (42.2, 53.0)	4.598	0.04	−0.662

## Discussion

The relationship between body composition and upper limb physical fitness is investigated by both the 4-year longitudinal survey (study 1) and the factorial experimental study (study 2). Study 1 analyzed BMI and pull-up performance data of male university students from 2015 to 2018 using Hierarchical Linear Model. The results showed that both the number of pull-ups and their grades showed a declined development tendency, while BMI showed an increased tendency, which supports Hypothesis 1a. The quadratic BMI could predict the number of pull-ups in the following year, suggesting that healthier body composition improved better upper physical fitness, which supports Hypothesis 1b. In study 2, two groups of participants had different body composition (measured by high/low total body fat percentage and total lean body mass percentage) respectively, and their body composition was measured by grip strength, upper body strength and anaerobic power of upper limbs. Healthier body composition was associated with higher upper limb physical fitness, which supports Hypothesis 2.

These results are consistent with previous studies. A study found that the average BMI of the Chinese population increased as the dietary patterns of urban and rural residents in China changed and their physical activity decreased (Wang et al., 2021). A systematic review also found that the strength and endurance of students in 14 countries were declining and that physical strength of students in China increased from 1985 to 1995 and declined between 1995 and 2014 (Masanovic, et al., 2020b). Other studies also found that the increase in body weight is one of the main reasons for the decline in physical fitness (Tittlbach et al., 2017; Bulbrook et al., 2021), even during the COVID-19 pandemic (Basterfield et al., 2022).

This phenomenon may be due to lifestyle changes. Studies have found that students are experiencing changing lifestyles (Masanovic, et al., 2020b), like a sedentary lifestyle (Morales-Demori et al., 2016) with the lack of physical activity and the increasing of screen time (Venckunas et al., 2017). Studies in China also found the decline in fitness level was caused by lifestyle changes, such as prolonged media use and increased consumption of fast food (Ao et al., 2019; Dong et al., 2019). These results suggest unhealthy exercise habits (lack of exercise, long sedentary time, *etc.*) and diet structure (unhealthy food intake) may be important factors affecting body composition and physical fitness.

The results of study 2 not only replicating study 1’s result that body composition contributed to the physical fitness of upper limbs, but also indicated that body composition impacted three aspects of upper limb physical fitness (grip strength, drape height and anaerobic power). Finger strength and shoulder strap strength were evaluated by the handgrip strength test (Laffaye et al., 2014), the explosive force of the shoulder and back of the upper limbs was measured by Power Slap Test (Draper et al., 2011), and the endurance of the upper muscles was measured by anaerobic power (Lovell et al., 2013). If students want to improve their upper body strength with low body composition, in addition to reducing BMI and improving body composition, they can also design relevant training programs through these aspects to help students develop their upper body quality. In the future, self-service, personalized and diversified online training programs can be considered so that more students can learn and train through online videos.

For individual students, potential suggestions could include: pay attention to the overall improvement of upper body strength quality, not only to improve the pull-up test results but also to train around the whole elements of upper body strength quality, including maximum strength, strength endurance, explosive power, and coordination with the lumbar abdominal muscle group. In addition to the above training, lifestyle can also be considered (Ao et al., 2019; Dong et al., 2019). For example, a number of studies have found the transfer between a healthy diet and physical exercise; that is, improving healthy diet can also promote individual physical exercise (Duan et al., 2017; Liang et al., 2019). Meanwhile, in parenting, caregivers should be urged to increase their companionship with their children, especially in sports activities, and reduce their own and their children’s use of electronic media, which can further increase children’s sports activities and improve their overall physical and mental health.

For universities, in the content of physical education curriculum and extracurricular exercise, educators could increase exercises related to upper body strength, and the training content of upper body strength could be organic and unified, rich and diverse. For example, more attention and encouragement would be given to students whose physical fitness is reduced after entering college. Meanwhile, it is suggested that universities could set up more rich and interesting physical courses about improving upper limbs (like rock climbing classes), to improve students’ motivation in exercising upper limbs. Moreover, universities could give awards and gifts to students who have more sports activities about upper limbs.

There are some limitations to this study, and there are many potential directions for further research in the future. First, this study only included male college students. Although females’ pull-up performance is not measured in China at present, upper body strength is still an important factor of physical fitness for female individuals (Venckunas et al., 2017). Considering the presence of gender differences in BMI and physical fitness development (Zhang et al., 2018), future studies could include female individuals and investigate the similarities and differences of these results. Secondly, study 1 used pull-ups performances for the upper limb body quality. For this concern, study 2 further used 3 different tests for upper limb multidimensional indexes. In addition to a wider variety of upper limb physical fitness indicators, future studies can also include core and lower limb physical fitness and psychological measures (Masanovic, et al., 2020b), and further explore the developmental trajectory of overall physical and mental health among young adults. Lastly, future research can design tailored electronic and digital intervention programs for physical fitness (Watson et al., 2017), identify target populations for interventions, and promote the development of children and young adults’ physical and mental health.

## Conclusion

This study highlights the quadratic predictive effect of body composition on upper limb physical fitness. The results suggest that physical fitness gradually declines after male students entering college, and bad body composition has a negative impact on many aspects of physical fitness, like grip strength, drape height, and anaerobic power. Results from our studies help society and educators to understand why students’ physical fitness continues to decline and provide a theoretical basis for future targeted intervention studies. Future researchers should pay more attention to students’ upper limb physical fitness. Through physical exercise, students’ upper arm muscle strength, upper arm shoulder, back explosive power and upper limb muscle endurance, and others upper limb physical fitness should be strengthened. Meanwhile, to enhance students’ body composition and physical fitness, future research could focus on healthy diets, parent-child relationship, and integrated design of physical education curriculum, for further promotion of students’ overall development of the physical and mental health.

## Data Availability

The raw data supporting the conclusions of this article will be made available by the authors, without undue reservation.

## Abbreviations

HLM, Hierarchical Linear Model; BMI, Body-Mass-Index.
