# Supplementary figures and images for: Relationship between body composition and upper limb physical fitness among Chinese students: 4-Year longitudinal follow-up and experimental study
Source: Front Physiol. 2023 Mar 3;14:1104018. doi: 10.3389/fphys.2023.1104018 (PMC10020539; doi:10.3389/fphys.2023.1104018)

## 4

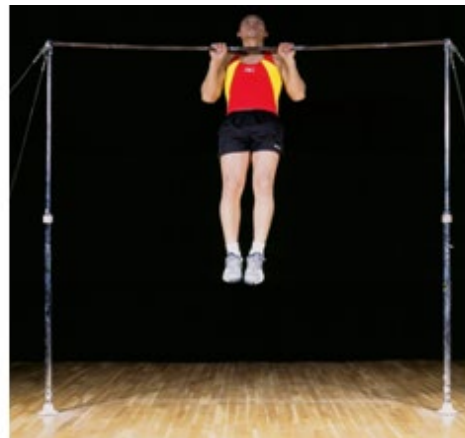

8

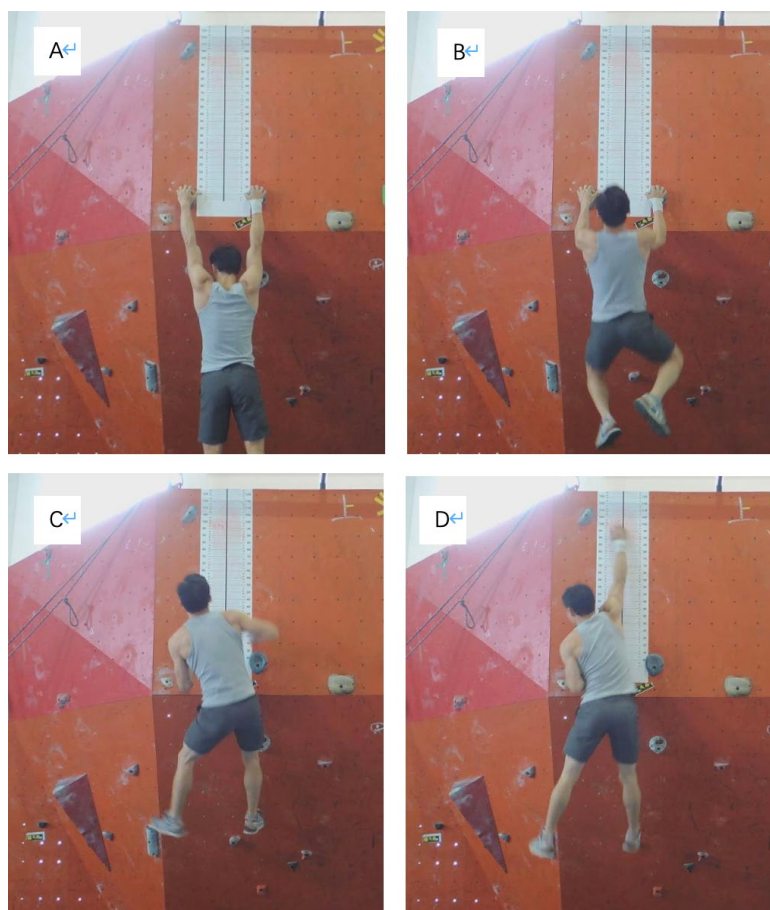

1

Supplement: Supplementary file 1 [file Image1.pdf]
